# Analysis of IoT-Related Ergonomics-Based Healthcare Issues Using Analytic Hierarchy Process Methodology

**DOI:** 10.3390/s22218232

**Published:** 2022-10-27

**Authors:** Hemant K. Upadhyay, Sapna Juneja, Ghulam Muhammad, Ali Nauman, Nancy Awadallah Awad

**Affiliations:** 1 BM Institute of Engineering and Technology, Sonepat 131001, India; 2 KIET Group of Institutions, Delhi NCR, Ghaziabad 201206, India; 3 Department of Computer Engineering, College of Computer and Information Sciences, King Saud University, Riyadh 11543, Saudi Arabia; 4 Department of Information and Communication Engineering, Yeungnam University, Gyeongsan 38541, Korea; 5 Department of Computer and Information Systems, Sadat Academy for Management Sciences, Cairo 11742, Egypt

**Keywords:** healthcare, Internet of Things (IoT), ergonomics, analytic hierarchy process

## Abstract

The objective of the present work is for assessing ergonomics-based IoT (Internet of Things) related healthcare issues with the use of a popular multi-criteria decision-making technique named the analytic hierarchy process (AHP). Multiple criteria decision making (MCDM) is a technique that combines alternative performance across numerous contradicting, qualitative, and/or quantitative criteria, resulting in a solution requiring a consensus. The AHP is a flexible strategy for organizing and simplifying complex MCDM concerns by disassembling a compound decision problem into an ordered array of relational decision components (evaluation criteria, sub-criteria, and substitutions). A total of twelve IoT-related ergonomics-based healthcare issues have been recognized as Lumbago (lower backache), Cervicalgia (neck ache), shoulder pain; digital eye strain, hearing impairment, carpal tunnel syndrome; distress, exhaustion, depression; obesity, high blood pressure, hyperglycemia. “Distress” has proven itself the most critical IoT-related ergonomics-based healthcare issue, followed by obesity, depression, and exhaustion. These IoT-related ergonomics-based healthcare issues in four categories (excruciating issues, eye-ear-nerve issues, psychosocial issues, and persistent issues) have been compared and ranked. Based on calculated mathematical values, “psychosocial issues” have been ranked in the first position followed by “persistent issues” and “eye-ear-nerve issues”. In several industrial systems, the results may be of vital importance for increasing the efficiency of human force, particularly a human–computer interface for prolonged hours.

## 1. Introduction

### 1.1. Background

Future healthcare could be more effective because to IoT. Interoperability, machine-to-machine connectivity, and data transfer enabled by IoT have increased productivity in the healthcare sector. Ergonomics is an applied science that focuses on creating and organizing the objects that people use in order to maximize the effectiveness and security of their interactions. Ergonomics can lessen the likelihood of workplace illnesses like hearing loss brought on by noise exposure at work, aches, pains, and injury to the wrists, shoulders, and back. The analytic hierarchy process (AHP) approach is a reliable and adaptable multi criteria decision making (MCDM) tool. The AHP compares various possibilities and picks the best one using a combination of arithmetic and psychology. One such method that can combine quantitative and qualitative features while also structuring problems is the AHP. The AHP uses pairwise comparison questions to elicit judgments about the relative importance of each pair of qualities, as well as preferences for each pair of alternatives with regard to each attribute.

### 1.2. Problem

Ergonomic IoT-related healthcare issues are required to be identified and relatively compared. Ergonomic IoT-related healthcare issues are required to be categorized into different categories. MCDM techniques may be utilized for ranking ergonomic IoT-related healthcare issues.

### 1.3. Proposed Solution

It is pertinent to cause the youthful ages to achieve the skill of getting data through a few assets and usage of the got data. The trademark of Internet for everyone has been embraced by every one of the social orders meaning to turn into a data society. Instructors should give a superior chance to understudies, cause understudies to accomplish better encounters, advantage from the chances in the time of the web in learning exercises and increment their expertise in their particular discipline. Data and communication technologies are responsible for a tutor and managing in preparation of all educators and understudies’ in scholastic institutions [1]. A detailed study is needed to be focused on technical industrial growth along with human factors and ergonomics considerations in the production context, as well as computer use [2]. IoT emerged in the past decade and now we are talking about lots and lots of gadgets, machines, buildings, and more, that connect to the web for connectivity and real-time data extraction [3]. It is observed that while using IoT, individuals are neglecting physical health by ignoring some rules of ergonomics or mental health, working chaotically without a program, neglecting time management, thereby overlooking the use of proper ergonomics, increasing the risk of injury and health problems.

The presented work suggests the assessment of ergonomics based IoT related healthcare issues in four major classes: excruciating issues, eye-ear-nerve issues, psychosocial messes, and persistent problems and shows the utilization of the application of multi-criteria decision-making technique AHP for the choice and prioritization of these standards. As far as dynamic utilization of data innovation in schooling internet learning assets are quickly creating. This interaction additionally included prestigious higher instructive foundations (for example, the Massachusetts Institute of Technology) [4]. Internet learning over the most recent twenty years has added to another time of instruction [5]. Internet learning application offers a wide arrangement of uses that oversee assorted kinds of media to convey professional preparation [6]. These frequently supplement conventional strategies empowering understudies to draw in from any spot with their learning through different materials rather vis-à-vis educating delivery [7].

### 1.4. Organization of the Paper

Section 2 outlines a review of relevant literature and recent past work has been discussed. Section 3 shows the methodology used, i.e., AHP, a popular MCDM technique, and its steps with formulae. Section 4 is devoted to a brief introduction to the work related to ergonomics-based IoT-related healthcare issues and the concerned work has been revisited. Section 5 reports the results and interpretation of the findings. Pair-wise comparison matrix and normalized pair-wise comparison matrix have been formed as per the data collected. Further criteria weights and consistency has been calculated to rank all twelve ergonomic-related issues in categories and subcategories. Finally, Section 6 provides concluding remarks on the paper and offers prospects for future work in this research.

## 2. Related Works

Digital wellness is a new term that refers to the lack of balance that we may feel when using mobile devices [8]. The idea of digital well-being has evolved as a fresh explanation for the everyday inundation of information and social networking alternatives [9]. Many technology businesses have added healthcare capabilities for measuring time spent and promoting pauses in use. The advocacy of technological abstinence is becoming more popular. Many people’s lives are enriched by digital technology, and digital wellness characteristics are arguably better than abstinence [10]. Researchers must consider the relationship between digital media use and well-being because conceptual models appear inadequate to capture the complexity of individuals’ relationships with digital media, and empirical approaches are criticized for lacking methodological rigor [8].

For the betterment of users’ relationships with technological advancements, the Human-Computer Interface community has been creating tools for “healthcare” [11]. The authors had done a review of the characteristics of more than forty healthcare applications analyzing more than one thousand user reviews of applications. Based on a few weeks’ long observation of a particular application with more than thirty users; they concluded that healthcare applications are unable to the promotion of the establishment of new practices. Healthcare applications have not been influential to assist users in practicing changes in conduct with smartphones. Authors explored to overcome the disadvantages of pure self-monitoring approaches, healthcare solutions that are more anchored in habit formation and social support theories are being developed [12]. The authors have created a theoretical model of healthcare that takes into consideration the dynamic and complicated character of people’s interactions with mobile technology, overcoming the conceptual and methodological constraints of previous methods. Healthcare, according to their concept, is an experienced state of optimum connection and disconnection that is dependent on several people-, device-, and context-specific elements. The authors hypothesized that the constellations reflect healthcare routes, and that the efficacy of healthcare treatments is determined by how disruptive they are to these pathways. The authors went on to say that experiences are a result of interactions between persons, devices, and contexts that can be modeled and empirically investigated as pathways in a dynamic system of well-being [8].

The authors have been focusing on several healthcare features and their work has facilitated speculative design for health care of the community for ensuring their designs for mitigating negative influences from technology. The authors may embrace several complexities in designing healthcare to reduce the harm caused by digital technology in the lives of users [11]. The authors presented a study that develops a theoretical proposal regarding attention. The presented work by authors has connected the design of social media to its influences on health care. The “theoretical construction of attention” methodology has revealed the consequences of social media consumption on human attention. The excess consumption of social media has become a threat that develops a set of repercussions for healthcare. The methodological advancements like data collection by brain monitoring along with eye tracking methodologies demonstrate the influences that social media consumption has on people’s attention [13].

Digital self-control tools (DSCTs) are external applications that facilitate the users in self-regulating the technique used with interventions such as lockout mechanisms and timers [14]. However, DSCTs primarily focus on interactions between users and a single device at a time, although most people use multiple devices at the same time [15]. The authors reviewed hundreds of DSCTs and observed that healthcare problems are not limited to smartphones but the simultaneous usage of several devices [14]. The authors analyzed more than three hundred applications for identifying common core design characteristics of existing techniques for digital self-control provide They also used an integrated dual systems model of self-regulation to organize and evaluate the design elements discovered [16].

Benmoussa et al. applied the MCDM technique, with four prime categories consisting of 16 attributes for analysis of the ergonomic evaluation of the information systems. For the validation of their work, a comprehensive analytical study was carried out at the university of Morocco [17]. Ayyildiz et al. focused on assessing the influence of anthropometric and environmental barriers in the distance learning process. The data are collected via a questionnaire filled by 100 university students who attend the ergonomics course online. Thirty-nine sub-factors are evaluated under five titles and the most important factors are determined [18]. Azleen et al. investigated seven challenges related to online learning among higher education scholars in Malaysia. The online interviewing was done for more than a hundred students from more than fifteen institutions [19].

Koppiahraj et al. identified and evaluated the ergonomic factors affecting the productivity of leather garment-based SMEs. In 3 emerging categories, 20 factors had been recognized for assessing symmetrical influence in 5 leather garment organizations. A sensitivity analysis had been carried out to validate the robustness of the findings [20]. The experimental work considered a block-based modification of the AHP through a realistic case study for the construction sector. A group of two key performance indicators (KPIs) compatible for comparison and developed two consistent AHP matrices through questionnaires by using a voting procedure. The weight of each KPI was evaluated by incorporating block based modified AHP as proposed in the work [21].

## 3. Methodology

Operations research’s MCDM assesses numerous, contradictory criteria in decision-making (in daily life, business, government, medicine). A solution requiring agreement is produced by the MCDM technique, which integrates an alternative’s performance across several, conflicting, qualitative and/or quantitative criteria. The use of computational methods that combine many criteria and order of preference in evaluating and choosing the best option among many possibilities based on the desired outcome is known as the application of MCDM theory. Fuzzy decision-making strategies can successfully handle the imprecision and ambiguity that are frequently present in decision-making. The theoretical and applied facets of MCDM have been the subject of extensive research in recent years.

The AHP, a dependable MCDM tool, is a decomposition of a complicated issue into simpler parts, based upon experts’ opinions, on the premise of a pairwise matrix of comparison. For developing different priority values for all criteria, the comparison of two possible options is required to show and establish the preference [22].

The AHP method uses a consistency index to calculate consistency. The AHP allows users to examine the relative weighting of many alternatives concerning specified parameters. The AHP has found applications in a variety of sectors where prioritization or foresight is necessary. Analytic hierarchy process allows for the spontaneous evaluation of comparative bias of various criteria against particular criteria. The power of the AHP to ponder criteria and alternatives makes it an effective method for industry-based applications [23].

### Analytic Hierarchy Process

The AHP is used in various aspects of life, including government policymaking, research and development, academic activities, corporate decisions, defense, and many other areas where decisions are made based on choice, preference, or prediction [24]. The AHP i.e., analytic hierarchy process is a multi-criteria decision-making technique, originally proposed by Saaty (1980) facilitating the decision-maker to solve a complicated problem [25]. The AHP approach is useful in a variety of decision-making situations because it uses a reciprocal decision matrix created from paired comparisons [26].

The AHP, having both qualitative and quantitative techniques, is instrumental in numerous applications in science and engineering [27]. The valuations are based on modest pair-wise assessments of the components, and AHP rank scales them and blends them into a cumulative hierarchy structure [28]. Determining the sample size is very crucial in the AHP method [29]. The priority vectors against measures from specified scales should be normalized by dividing their total; if two vectors become nearly the same, the AHP model’s findings indicate the model’s validation [30].

The AHP is a flexible strategy for organizing and simplifying complex MCDM concerns by disassembling a compound decision problem into an ordered array of relational decision components (evaluation criteria, sub-criteria, and substitutions). Following the identification of AHP’s hierarchy structure, the AHP can be called using the procedures below [31]. In the AHP, pair-wise comparison approaches are frequently utilized to deal with criteria decisions [32].

The AHP evaluates different criteria pairwise while preserving a measure of one parameter’s predominance over the other. The Saaty scale (1980) quantitatively grades between one and nine, with one representing equal importance and nine representing crucial importance [33]. An improved version of analytic hierarchy process has also been introduced, which can greatly minimize the number of desired comparisons, making it suitable for company benchmarking in competitive environments such as the real estate industry [21]. The AHP is an effective multi-criteria decision-making technique with multi-level structure modeling of a set of factors [34]. AHP methodology has got applications in complex problems of various disciplines like engineering, sciences, and management [35]. Multi-sensor data fusion (MDF) is one of the most often utilized techniques for extending network lifetime [36].

The AHP method is to convert the decision-based problems into a set of hierarchies, to compute criterion and sub-criterion, where all components in the hierarchy of criteria are required not to be dependent on any other component [37]. The AHP has proven to be a powerful systematic, scientific, and dependable MCDM technique that can handle a wide range of input data. It is a dissection of a complex problem into a few components and the application of experts’ perspectives to the premise of a pairwise matrix of comparison [38].

The AHP number scale, famous as the Saaty scale, is a 1 to 9 scale that represents 1 for “equal importance” and 9 for “extreme importance”. Analytic hierarchy process is the set of following steps, i.e., it is a step-by-step procedure [31]. All the steps have been mentioned below:

Step 1: Generalized pairwise comparison matrix (size n × n) (while n will be the number of components of the particular level) [n(n − 1) number of judgments will be prerequisite to complete the PWCM].

Step 2: Development of a pairwise comparison matrix (having all diagonal elements unity).

Step 3: Development of a pair-wise comparison matrix (considering the priorities of decision elements as shown in Table 1 (reciprocals are assigned automatically in each pair-wise comparison).

Step 4: Construction of normalized pair wise comparison matrix.

Step 5: Hierarchy synthesis to determine the weights of the criteria.

Step 6: Construction of consistency calculation matrix.

Step 7: Computation of weighted sum values.

Step 8: Eigen values λ = weighted sum value/criteria weight.

Step 9: Principal Eigenvalue is the mean of all Eigenvalues λmax =∑ λn.

Step 10: Consistency index is the value CI = (λ_max_ − n)/(n − 1).

Step 11: The dominant quantum values of the random index (RI) are given in Table 2.

Step 12: CR may be calculated to ensure the consistency of pair-wise comparisons; consistency ratio CR = ; A consistent CR has a value of 0.1 or less and is acceptable; else inconsistent [36].

Table 1 shows the numeral value representation of the relative importance of issues to develop a pair-wise comparison matrix with consideration of the priorities of decision elements. Further, the reciprocals are assigned automatically in each pair-wise comparison.

Table 2 represents the numeral values of random index for a different number of issues/criteria in AHP methodology.

Table 3 shows the suggested Hierarchy for all the criteria.

## 4. Proposed Model

The ergonomics-based IoT-related healthcare issues have been categorized as excruciating issues, eye-ear-nerve issues, psychosocial issues, and persistent issues proposing the model in the hierarchy order. A total of twelve IoT-related ergonomics-based healthcare issues have been abbreviated from ISS1 to ISS12. To obtain the prime purpose of our developed hierarchy, which is the evaluation of IoT-related ergonomics-based healthcare issues, 12 issues are hierarchal settled in 2 levels as mentioned in Table 3.

New technical advancements have unanticipated impacts on workers and computer users from an ergonomics point of view. The literature surveyed indicated the insufficiency from the perspective of ergonomics issues [2].

### 4.1. Excruciating Issues

Excruciating Issues are a set of conditions influencing the muscles and joints [39,40]. These can affect back. This is typically a direct result of a helpless working stance and is related to neck pain and shoulders too [41].

#### 4.1.1. Lumbago (Lower Backache)

Utilization of PC and stationary sitting might foster an effect on the lower backache of users, because of inappropriate postures [42]. Lower backache is experienced by PC user who needs to operate on the keyboard but in a passive form [43]. Lower backache at the same time makes impacts different muscles developing the pain [44].

#### 4.1.2. Cervicalgia (Neck Ache)

A very solid connection between passive sitting and pain in the neck exists noticing an affirmative relationship between neck flexion with pain in the neck [45]. There is a bigger descending slant for connection along with the minor risk of issues of the neck [46].

#### 4.1.3. Shoulder Pain

Lozano et al. [47] concluded that interactions with touch-screen tablets affect the whole shoulder system. Offensive poses (i.e., flexion and abduction) in the shoulder part are based on ergonomic issues [48]. Marcus et al. [46] observed that the inner elbow angle is the posture variable which proved an enhanced danger for Issue.

### 4.2. Eye-Ear-Nerve Issues

#### 4.2.1. Digital Eye Strain

Hayes et al. [49] investigated that environmental variable is generally based on eye strain that dominantly affects personal convenience [50].

#### 4.2.2. Hearing Impairment

Ongoing research has seen that more than globally one billion young people might be at risk for hearing impairment due to insecure listening practices [51]. There is a considerable likelihood of the development of reflexes in the internal ear [52,53].

#### 4.2.3. Carpal Tunnel Syndrome

CTS usually comes in the scenario with a nerve compression problem [54] with feelings like numbness and pain within that concerned part of the body [55,56,57]. Thomsen et al. [54] explored that biological and mechanical considerations like repetitive odd postures enhance the danger of carpal tunnel syndrome with the enhancement of carpal tunnel stress. The pressure associated with the keyboard is a risk issue for carpal tunnel syndrome [58]. Keyboard users display a range of convenient postures [59] being movement less while typing [60]. Odd typing postures are recognized as a danger factor for the creation of CTS symptoms [61,62]. Several papers have shown that an enhanced case of carpal tunnel syndrome may be based on the greater use of the mouse of the computer [63].

### 4.3. Psychosocial Issues

Managing various psychosocial considerations are different from psychosocial ones. Sometimes it could be extremely difficult to address psychosocial circumstances straightforwardly [64].

#### 4.3.1. Distress

The meaning of the word stress might be a non-particular reactive response to the stress-causing thing or person, comprising of a few physiological responses [65]. A few of the structures show that non-favorable psychosocial circumstances become a reason for the distress that is supposed to improve the danger of musculoskeletal side effects [66]. A few investigations point out the dominance of distress [67].

#### 4.3.2. Exhaustion

To detect the sign of ergonomic issues, a survey [68] was performed to acknowledge the part of the body where computer user experiences indications of weakness [69]. Passive sitting is related to internal abdominal muscle exhaustion that may impair spinal stability [70].

#### 4.3.3. Depression

Sedentary sitting ahead of a computer screen for several hours daily may drastically enhance the danger of depression and insomnia [71]. Depression is among the initial occupational health symptoms [72]. Studies about the dangers of computers in ergonomics-based IoT-related healthcare issues have concluded that they can also affect wellness and health.

### 4.4. Persistent Issues

#### 4.4.1. Obesity

The condition is outlined by any person’s BMI. BMI, i.e., body mass index, is the ratio between mass in kilograms and square of height in meters. Continuous several hours sitting in front of screens is a component of daily life and is connected with obesity [73].

#### 4.4.2. High Blood Pressure

Several investigations have exhibited relationships between critical web use and medical problems like high blood pressure [74]. Screen time has a clear connection with high blood pressure irrespective of the physique of any human. Hindering passive sitting with computer use (light walking or basic exercises) may effectively lessen blood pressure [75,76].

#### 4.4.3. Hyperglycemia

American Hyperglycemia Association issued a statement in 2016 suggesting the reduction in the passive sitting of computer users [77]. The continuous number of hours of everyday computer sitting might be connected with unwell being results in hyperglycemia patients [78,79,80]. The authors in [6] showed a connection between hyperglycemia and passive sitting with the computer.

## 5. Results and Discussion

For validating the suggested model, a web-based Google-form questionnaire was circulated to get raw data input from a variety of respondents belonging to several spheres of life like academicians, students, computer users, medical practitioners, biotechnologists, technocrats for creating a pairwise comparison matrix for analytic hierarchy process methodology for assessment and computation of relative importance of ergonomics-based issues in online learning. A total of more than one hundred fifty responses from several parts of India have been collected through an online web-based form from experts of different age groups and professional positions. We present the development of the pairwise comparison of the IoT-related ergonomics-based healthcare issues in Table 4.

Normalization of the pair-wise comparison matrix of the IoT-related ergonomics-based healthcare issues has been presented in Table 5.

Criteria weights for IoT-related ergonomics-based healthcare issues have been computed and expressed in Table 6.

Calculated values for consistency of IoT-related ergonomics-based healthcare issues have been represented in Table 7.

The computation of principal Eigenvalues for IoT-related ergonomics-based healthcare issues has been shown in Table 8.

The calculation of principal Eigenvalue and consistency index (AHP calculation) is:
λmax = (λ1 + λ2 + λ3 + λ4 + λ5 + λ6 + λ7 + λ8 + λ9 + λ10 + λ11 + λ12)/12 = 12.830Consistency Index = [λmax − n]/[n − 1]C. Index = (12.830 − 12)/(12 − 1) = 0.830/11 = 0.0755


The calculation of the consistency ratio (AHP calculation) is as below:

Consistency Ratio = Consistency Index/Random Index = 0.0755/1.535 = 0.0492 < 0.1

The consistency ratio must be a maximum of 0.1 for ensuring judgment consistency.

Table 9 depicts the priority computed using the AHP methodology and the rank of the several sub-criteria. It can be noticed that “distress” is the most dominant IoT-related ergonomics-based healthcare issue as shown in Table 9.

**Figure 1:**
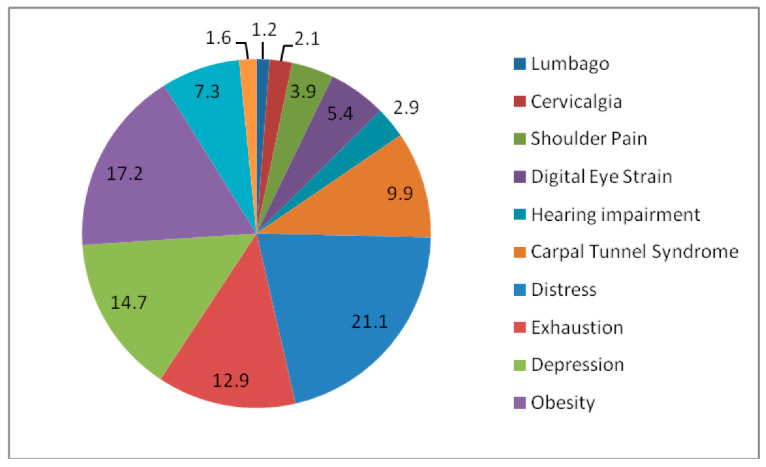
Priority (%) values of different IoT-related ergonomics-based healthcare issues.

Figure 1 shows the relative weight ages of all twelve issues. Lumbago is the least influential IoT-related ergonomics-based healthcare issue among the chosen set.

Based upon the numerical values indicated in Table 10, psychosocial issues got the first rank, and the second rank was achieved by persistent issues.

**Figure 2:**
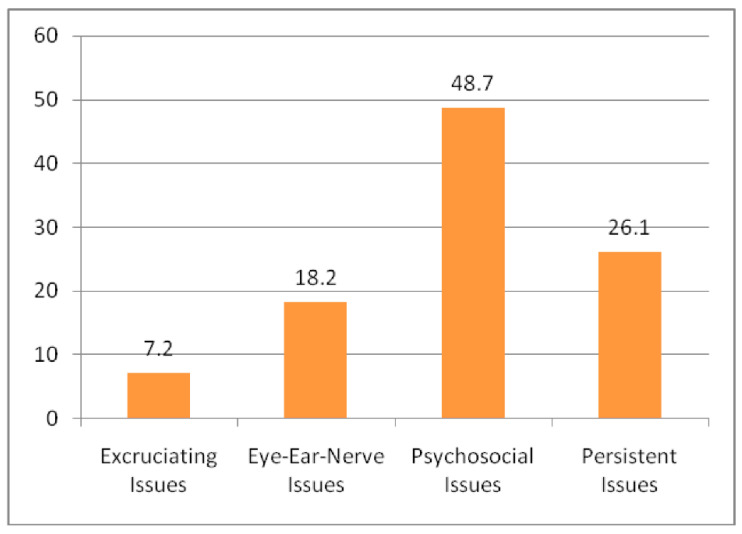
Category wise influence in IoT-related ergonomics-based healthcare issues.

As shown in Figure 2, excruciating issues are the least influencing category followed by the eye-ear-nerve issues category.

The outcome of the work by some of the similar efforts has been summarized in Table 11 below.

## 6. Conclusions

### 6.1. Concluding Remarks

The presented research work has developed an MCDM system for assessing IoT-related ergonomics-based healthcare issues by applying AHP technology. A total of twelve IoT-related ergonomics-based healthcare issues have been recognized and distress has proven itself the most critical IoT-related ergonomics-based healthcare issue, followed by obesity, depression, and exhaustion. These IoT-related ergonomics-based healthcare issues are in four categories, out of those, “psychosocial issues” has been ranked first, followed by “persistent issues” and “eye-ear-nerve issues”.

### 6.2. Limitations

The future scope of work can be the development of a similar structure with the application of other MCDM techniques available like TOPSIS (a technique for order performance by similarity to ideal solution), decision making trial and evaluation laboratory (DEMATEL), etc. Further work may be for developing a similar model with the application of other multiple-criteria decision-making methods. Modern MCDM techniques can be utilized to deal with complicated issues related to ergonomics.

## Figures and Tables

**Table 1:** Comparison scale in AHP.

Value	Category	Explanation
1	Equally dominant	Two actions endorse likewise for an objective
3	Feeble dominance of one over another	Familiarity and evaluation partially weigh an action to the other
5	Indispensable or compulsorily dominant	Familiarity and evaluation strongly weigh an action to the other
7	Established dominance	An action is predominantly biased, and its authority is validated in practice
9	Complete dominance	The indication is biased from one action to the other action, this is the maximum feasible degree of conformance
2, 4, 6, 8	Intermediary tenets among the two contiguous judgments	When there is a need for some negotiation

**Table 2:** Random index (RI) values for *N* criteria.

N	3	4	5	6	7
RI	0.58	0.90	1.12	1.24	1.32
N	8	9	10	11	12
RI	1.41	1.45	1.49	1.51	1.535

**Table 3:** The suggested hierarchy.

Criteria	Sub Criteria	Issue
Excruciating Issues	Lumbago	ISS1
	Cervicalgia	ISS2
	Shoulder Pain	ISS3
Eye-Ear-Nerve Issues	Digital Eye Strain	ISS4
	Hearing impairment	ISS5
	Carpal Tunnel Syndrome	ISS6
Psychosocial Issues	Distress	ISS7
	Exhaustion	ISS8
	Depression	ISS9
Persistent Issues	Obesity	ISS10
	High Blood Pressure	ISS11
	Hyperglycemia	ISS12

**Table 4:** The pair-wise comparative matrix of the IoT-related ergonomics-based healthcare issues.

Issue	ISS1	ISS2	ISS3	ISS4	ISS5	ISS6	ISS7	ISS8	ISS9	ISS10	ISS11	ISS12
ISS1	1	0.33	0.20	0.17	0.25	0.13	0.11	0.11	0.11	0.11	0.14	0.50
ISS2	3	1	0.33	0.25	0.50	0.17	0.14	0.14	0.14	0.14	0.20	2
ISS3	5	3	1	0.50	2	0.25	0.20	0.20	0.20	0.20	0.33	4
ISS4	6	4	2	1	3	0.33	0.25	0.25	0.25	0.25	0.50	5
ISS5	4	2	0.50	0.33	1	0.20	0.17	0.17	0.17	0.17	0.25	3
ISS6	8	6	4	3	5	1	0.33	0.50	0.50	0.33	2	7
ISS7	9	7	5	4	6	3	1	3	2	2	4	8
ISS8	9	7	5	4	6	2	0.33	1	0.50	0.50	3	8
ISS9	9	7	5	4	6	2	0.50	2	1	0.50	3	8
ISS10	9	7	5	4	6	3	0.50	2	2	1	3	8
ISS11	7	5	3	2	4	0.50	0.25	0.33	0.33	0.33	1	6
ISS12	2	0.50	0.25	0.20	0.33	0.14	0.13	0.13	0.13	0.13	0.17	1
Sum	72	49.83	31.28	23.45	40.08	12.72	3.91	9.83	7.33	5.66	17.59	60.5

**Table 5:** The normalized pair-wise comparison of the IoT-related ergonomics-based healthcare issues.

Issue	ISS1	ISS2	ISS3	ISS4	ISS5	ISS6	ISS7	ISS8	ISS9	ISS10	ISS11	ISS12
ISS1	0.014	0.007	0.006	0.007	0.006	0.010	0.028	0.011	0.015	0.019	0.008	0.008
ISS2	0.042	0.020	0.011	0.011	0.013	0.013	0.036	0.014	0.019	0.025	0.011	0.033
ISS3	0.069	0.060	0.032	0.022	0.050	0.020	0.051	0.020	0.027	0.035	0.018	0.067
ISS4	0.083	0.080	0.065	0.043	0.075	0.026	0.064	0.025	0.034	0.044	0.028	0.083
ISS5	0.056	0.040	0.016	0.014	0.025	0.016	0.043	0.017	0.023	0.030	0.014	0.050
ISS6	0.111	0.120	0.129	0.130	0.125	0.079	0.084	0.051	0.068	0.058	0.111	0.117
ISS7	0.125	0.140	0.161	0.174	0.150	0.236	0.256	0.305	0.273	0.353	0.222	0.133
ISS8	0.125	0.140	0.161	0.174	0.150	0.157	0.084	0.102	0.068	0.088	0.167	0.133
ISS9	0.125	0.140	0.161	0.174	0.150	0.157	0.128	0.203	0.136	0.088	0.167	0.133
ISS10	0.125	0.140	0.161	0.174	0.150	0.236	0.128	0.203	0.273	0.177	0.167	0.133
ISS11	0.097	0.100	0.097	0.087	0.100	0.039	0.064	0.034	0.045	0.058	0.056	0.100
ISS12	0.028	0.010	0.008	0.009	0.008	0.011	0.033	0.013	0.018	0.023	0.009	0.017

**Table 6:** The criteria weights of the IoT-related ergonomics-based healthcare issues.

	ISS1	ISS2	ISS3	ISS4	ISS5	ISS6	ISS7	ISS8	ISS9	ISS10	ISS11	ISS12
Criteria Weights	0.012	0.021	0.039	0.054	0.029	0.099	0.211	0.129	0.147	0.172	0.073	0.016

**Table 7:** The consistency calculation matrix for IoT-related ergonomics-based healthcare issues.

Issue	ISS1	ISS2	ISS3	ISS4	ISS5	ISS6	ISS7	ISS8	ISS9	ISS10	ISS11	ISS12
ISS1	0.012	0.007	0.008	0.009	0.007	0.013	0.023	0.014	0.016	0.019	0.010	0.008
ISS2	0.036	0.021	0.013	0.014	0.015	0.017	0.030	0.018	0.021	0.024	0.015	0.032
ISS3	0.060	0.063	0.039	0.027	0.058	0.025	0.042	0.026	0.029	0.034	0.024	0.064
ISS4	0.072	0.084	0.078	0.054	0.087	0.033	0.053	0.032	0.037	0.043	0.037	0.080
ISS5	0.048	0.042	0.020	0.018	0.029	0.020	0.036	0.022	0.025	0.029	0.018	0.048
ISS6	0.096	0.126	0.156	0.162	0.145	0.099	0.070	0.065	0.074	0.057	0.146	0.112
ISS7	0.108	0.147	0.195	0.216	0.174	0.297	0.211	0.387	0.294	0.344	0.292	0.128
ISS8	0.108	0.147	0.195	0.216	0.174	0.198	0.070	0.129	0.074	0.086	0.219	0.128
ISS9	0.108	0.147	0.195	0.216	0.174	0.198	0.106	0.258	0.147	0.086	0.219	0.128
ISS10	0.108	0.147	0.195	0.216	0.174	0.297	0.106	0.258	0.294	0.172	0.219	0.128
ISS11	0.084	0.105	0.117	0.108	0.116	0.050	0.053	0.043	0.049	0.057	0.073	0.096
ISS12	0.024	0.011	0.010	0.011	0.010	0.014	0.027	0.017	0.019	0.022	0.012	0.016

**Table 8:** The calculation of the principal Eigenvalue.

Issue	Criteria Weight Age	Weighted Sum	λ = Weighted Sum/Criteria Weight Age
ISS1	0.012	0.146	12.167
ISS2	0.021	0.256	12.190
ISS3	0.039	0.491	12.590
ISS4	0.054	0.69	12.778
ISS5	0.029	0.355	12.241
ISS6	0.099	1.308	13.212
ISS7	0.211	2.793	13.237
ISS8	0.129	1.744	13.519
ISS9	0.147	1.982	13.483
ISS10	0.172	2.314	13.453
ISS11	0.073	0.951	13.027
ISS12	0.016	0.193	12.063

**Table 9:** Sub-criteria priority results.

Criteria	Sub Criteria	Priority	Rank
Excruciating Issues	Lumbago	0.012	XIIth
	Cervicalgia	0.021	Xth
	Shoulder Pain	0.039	VIIIth
Eye-Ear-Nerve Issues	Digital Eye Strain	0.054	VIIth
	Hearing impairment	0.029	IXth
	Carpal Tunnel Syndrome	0.099	Vth
Psychosocial Issues	Distress	0.211	Ist
	Exhaustion	0.129	IVth
	Depression	0.147	IIIrd
Persistent Issues	Obesity	0.172	IInd
	High Blood Pressure	0.073	VIth
	Hyperglycemia	0.016	XIth

**Table 10:** Criteria priorities results.

Criteria List	Priorities	Ranking
Excruciating Issues	0.072	4
Eye-Ear-Nerve Issues	0.182	3
Psychosocial Issues	0.487	1
Persistent Issues	0.261	2

**Table 11:** Comparison of our work with some recent contributions.

Ref.	Paper	Outcome	Present Work
[17]	Benmoussa et al. (2019)	The authors have implemented MCDM using the AHP methodology. There were 4 main categories comprising 16 attributes in all, to analyze the ergonomic evaluation of the information systems. A comprehensive analytical study was carried out at the university of Morocco to ensure the validity of their proposed research work.	The presented work is to assess IoT-related ergonomics-based healthcare issues using the popular MCDM technique named AHP. A group dialogue was performed for identifying ergonomics-based IoT-related healthcare issues and the twelve ergonomic Issues in four categories were compared and ranked.
[69]	Pant et al. (2022)	They provided a brief overview of the functional connections between the well-known consistency indices that have been established in the literature.	Our work has utilized AHP for ranking the IoT-related ergonomics-based healthcare issues and the categories.
[28]	Sinuany-Stern, Israeli and Bar-Eli, (2006)	The authors have made a prediction model for the evaluation of the ranking of eleven basketball teams by incorporating the Analytical Hierarchical Process, through six criteria for evaluation and valuable inputs from four field experts. Consistency tests were further performed using five criteria and three field experts. It was observed that the AHP model’s predictions displayed a significant correlation compared to the actual ranking when matched at the season’s end.	The present work is for analyzing IoT-related ergonomics-based healthcare issues with the use of a popular multi-criteria decision-making technique named the AHP.A total of twelve IoT-related ergonomics-based healthcare issues have been identified and kept in four major categories. These were ranked by applying AHP method for respective priority values.
[70]	Ruiz et al. (2021)	The AHP, a top multiple criteria decision-making (MCDM) technique, can be used to evaluate different plans for urban transportation in order to address shared objectives among municipalities.	Our work has utilized AHP for ranking the IoT-related ergonomics-based healthcare issues and the categories.
[21]	Foteinopoulos, Papacharalampopoulos and Stavropoulos, (2019)	This experimental work considered a block-based modification of the AHP through a realistic case study for the construction sector. A group of two KPIs compatible for comparison and developed 2 consistent AHP matrices through questionnaires by using a voting procedure. The weight of each KPI was evaluated by incorporating block-based modified AHP as proposed in the work.	The presented research work has developed a multi-criteria decision-making system for assessing IoT-related ergonomics-based healthcare issues by applying AHP technology. A total of twelve IoT-related ergonomics-based healthcare issues in four categories (excruciating issues, eye-ear-nerve Issues, psychosocial issues, and persistent issues), have been compared and ranked.
